# Effect of frailty, physical performance, and chronic kidney disease on mortality in older patients with diabetes : a retrospective longitudinal cohort study

**DOI:** 10.1186/s13098-022-00972-0

**Published:** 2023-01-17

**Authors:** Shuo-Chun Weng, Cheng-Fu Lin, Chiann-Yi Hsu, Shih-Yi Lin

**Affiliations:** 1 Department of Post-Baccalaureate Medicine, College of Medicine, National Chung Hsing University, Taichung, Taiwan; 2 Research Center for Geriatrics and Gerontology, College of Medicine, National Chung Hsing University, Taichung, Taiwan; 3 Center for Geriatrics and Gerontology, Division of Nephrology, Department of Internal Medicine, Taichung Veterans General Hospital, Taichung, Taiwan; 4 Institute of Clinical Medicine, School of Medicine, College of Medicine, National Yang Ming Chiao Tung University, Taipei, Taiwan; 5 Division of Occupational Medicine, Department of Emergency, Taichung Veterans General Hospital, Taichung, Taiwan; 6 Biostatistics Task Force of Taichung Veterans General Hospital, Taichung, Taiwan; 7 Center for Geriatrics and Gerontology, Division of Endocrinology and Metabolism, Department of Internal Medicine, Taichung Veterans General Hospital, Taichung, Taiwan

**Keywords:** Comprehensive geriatric assessment, Chronic kidney disease, Diabetes, Frailty, Handgrip strength, Mortality, Timed up and go test

## Abstract

**Background:**

Declined renal function is associated with physical function impairment and frailty in a graded fashion. This study aimed to examine the relationship between renal function, frailty and physical performance with mortality in older patients with diabetes, while also determining their combined effects on patient outcome.

**Methods:**

A retrospective longitudinal study was conducted in elderly patients with diabetes. Kidney disease staging was based on clinical practice guidelines of the International Society of Nephrology, and chronic kiney disease (CKD) was defined as urinary albumin to creatinine ratio (UACR) > 30 mg/g, persistent reduction in estimated glomerular filtration rate (eGFR) below 60 mL/min per 1.73 m^2^ or both. The modified Rockwood frailty index (RFI) was composed of cumulative health deficits, and physical function was determined by handgrip strength (HGS). Additionally, a timed up and go (TUG) test was assessed at baseline. Kaplan-Meier survival and Cox proportional hazard analyses were used to analyze the association between CKD, frailty, physical function and mortality.

**Results:**

For the 921 enrolled patients, their mean age was 82.0 ± 6.7 years. After a median 2.92 (interquartile range [IQR] 1.06–4.43) year follow-up, the survival rate was 67.6% and 85.5% in patients with and without CKD, respectively. The mortality hazard ratio (crude HR) with CKD was 5.92 for those with an RFI higher than 0.313 (95% CI 3.44–10.18), 2.50 for a TUG time longer than 21 s (95% CI 1.22–5.13), and 2.67 for an HGS lower than 10.57 kg in females or 20.4 kg in males (95% CI 1.12–6.37). After multivariate adjustment, the mortality hazard ratio for an RFI ≥ 0.313 was 5.34 (95% CI 2.23–12.80) in CKD patients, but not in patients without CKD. In subgroup analysis, patients experiencing CKD and frailty, or physical function impairment, had the lowest survival proportion followed by only frailty/declined physical function, only CKD, without CKD, and non-frailty/non-physical impairment.

**Conclusion:**

CKD, frailty and physical function impairment were all associated with an increased mortality risk in older patients with diabetes, while the combined effects of these 3 factors were seen on patient outcome.

**Supplementary Information:**

The online version contains supplementary material available at 10.1186/s13098-022-00972-0.

## Introduction

Over the past years, mortality and the incidence of cardiovascular outcomes have declined substantially amongst persons with diabetes; however, the health and economic burden surrounding chronic kidney disease (CKD) in patients with diabetes remains high [1, 2]. Diabetes continues to be the leading cause of end-stage renal disease (ESRD) worldwide, being the cause of ESRD in 24–55% of patients [3, 4]. Importantly, older patients with diabetes have a faster decline in eGFR per year, along with an increase in death risk [5]. In addition to CKD, older adults with diabetes are also at a greater risk of geriatric syndromes, including physical disability, cognitive impairment, frailty, falls, polypharmacy, and others [6]. Frailty is broadly accepted as a multidimensional syndrome manifesting as a decrease in physiological reserve, as well as increased susceptibility to numerous adverse outcomes [7]. In patients with diabetes associated with frailty, a higher risk of disability, hospitalization, and mortality has been seen [8, 9]. Additionally, several epidemiologic studies have reported that physical disability associated with diabetes may profoundly affect both qualities of life in the older population with diabetes as well as the disease prognosis [10].

Patients diagnosed with CKD are more likely to advance to the frailty stage due to chronic inflammation, insulin resistance and vascular calcification, resulting in a loss of musculoskeletal mass [11]. Furthermore, greater renal function decline is associated with more severe frailty and physical function impairment, which as a consequence contributes to adverse health events in patients with CKD, regardless of whether they are receiving dialysis or not [12, 13]. As both CKD and frailty are associated with diabetes, their effects and outcomes are important in patients with diabetes. However, there have been few previous studies which focused on combined CKD and frailty when predicting mortality in older people with diabetes. The aim of the present study was two-fold. First, we investigated whether CKD and frailty, along with physical performance parameters, were independent predictors of mortality in elderly adults with diabetes. And secondly, we explored the combined associations of these factors on mortality.

## Materials and methods

### Assembly of the cohort, study population, and the follow-up procedure

**Fig. 1:**
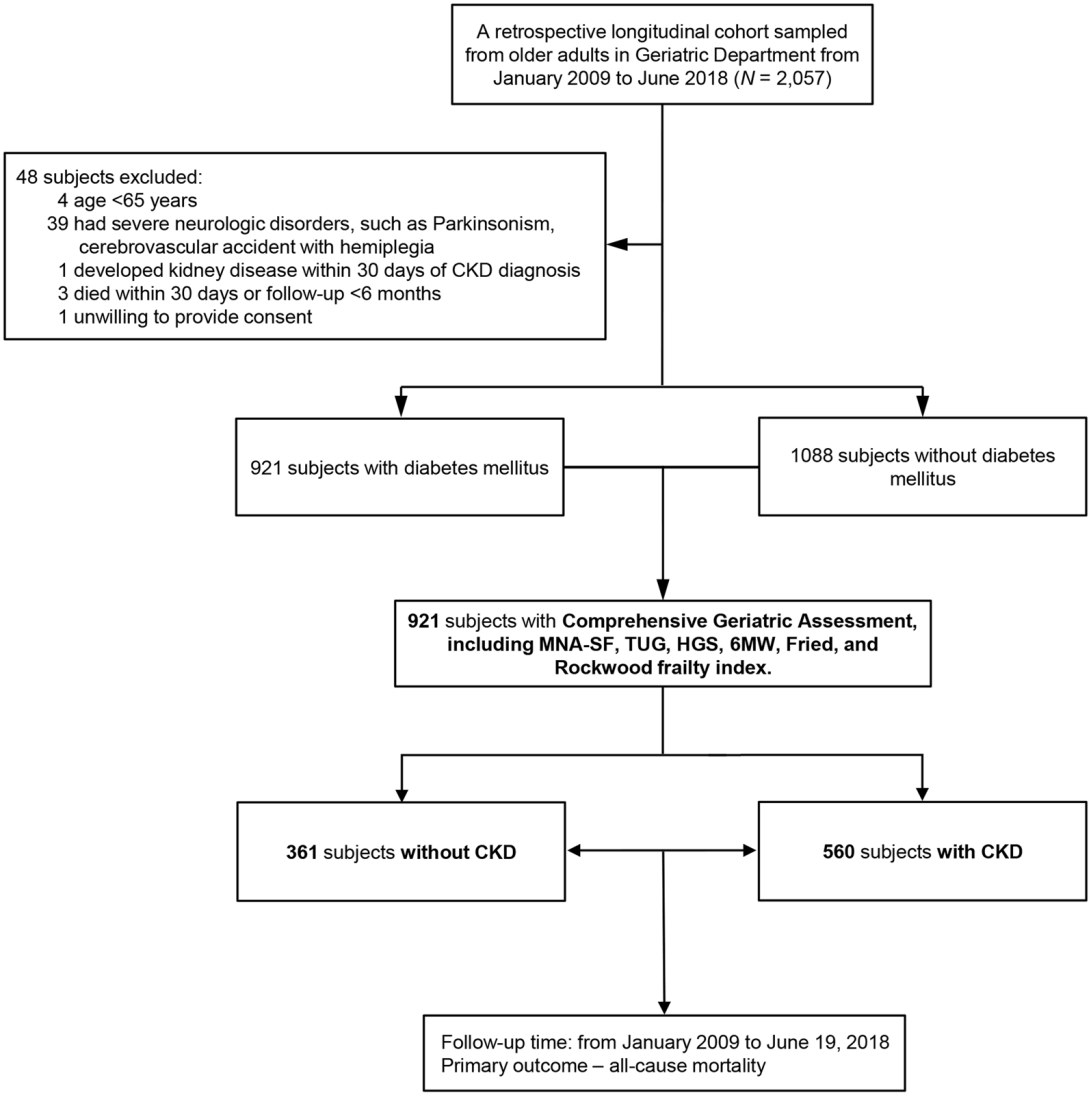
Flowchart presenting the selected participants. Five hundred sixty (560) patients had diabetes mellitus (DM) and chronic kidney disease (CKD), while 361 subjects had diabetes only, no CKD. *MNA-SF* mini-nutritional assessment-short form, *TUG* timed up-and-go test, *HGS* handgrip strength, *6 MW* 6-meter walking

This was a hospital-based retrospective longitudinal cohort study, and we enrolled 2057 patients ≥ 65 years from the case management care system of the Center for Geriatrics and Gerontology, Taichung Veterans General Hospital (TCVGH) with a study duration from Jan 2009 to June 2018. Diagnosis of diabetes mellitus (DM) was determined by the International Classification of Disease 9th version, Clinical Modification (ICD-9-CM) code of 250.x during outpatient visits, or at least once during in-patient care. The patients with age < 65 years, severe neurologic disorders, < 30 days of CKD diagnosis, death within 30 days or an inadequate follow-up length of < 6 months were excluded. Finally, 921 patients with DM were enrolled (Fig. 1). In this study, kidney disease staging is according to Kidney Disease: Improving Global Outcomes (KDIGO) guideline [14–16] with glomerular filtration rate (GFR) category (G1–G5; G1, eGFR ≥ 90; G2, eGFR 60–89; G3a, eGFR 45–59; G3b, eGFR 30–44; G4, eGFR 15–29; G5, eGFR < 15 ml/min/1.73 m^2^), albuminuria category with albumin/creatinine ratio (ACR) > 30 mg/g (A1–A3; A1, albuminuria < 30 mg/g; A2, albuminuria 30-300 mg/g; A3, albuminuria > 300 mg/g) [14], and a urine protein/creatinine (PC) ratio > 0.2 mg/g [15]. The diagnosis of CKD was defined by the following criteria: urinary albumin to creatinine ratio (UACR) > 30 mg/g, persistent reduction in estimated glomerular filtration rate (eGFR) below 60 mL/min per 1.73 m^2^, or both, for at least 3 months. Besides, in this study, CKD was also diagnosed with the ICD-9-CM code of 585.x that had been validated. Afterward, there were 560 diabetic subjects classified with CKD and 361 diabetic subjects without CKD with index dates defined as the day of receiving comprehensive geriatric assessment (CGA) for all participants. The enrolled subjects were generally followed up at the outpatient department and/or by telephone calls every 3 to 6 months until June 19, 2018. The patients with frailty were evaluated every 6 months; in the non-frail group, they were evaluated once a year. The retrospective study was approved by the Institutional Review Board of TCVGH (No. CE20293A, CF13015, CF13015-1, CF13015A-2, CF13015A-3) which approved the waiver for informed consent. All methods in this study were carried out by our institutional guidelines and regulations.

## Study variables

Clinical data, including demography, self-reported comorbidities (dementia, hypertension and hyperlipidemia), chronic obstructive pulmonary disease (COPD, ICD-9-CM codes of 491.X, 492.X, 493.22 and 496) [17], as well as chronic heart failure (CHF, ICD-9-CM codes of 428.0-428.9 and 402.91) were all validated by the ICD-9-CM codes. Additionally, a 2D echocardiogram and N-terminal pro-B-type natriuretic peptide (NT-proBNP) were obtained to diagnose and differentiate HF with preserved and reduced ejection fraction under a standard protocol (American Society of Echocardiography [ASE] or European Association of Cardiovascular Imaging [EACVI] protocol) [18]. In addition, body mass index, Charlson Comorbidity Index and laboratory tests were measured during visits to the in-patient and out-patient departments on the index date. Diabetic severity was measured using both serum glycated hemoglobin and fasting glucose. Diabetes medication, including oral antidiabetic agents (OADs) (α-glucosidase inhibitors, biguanides, meglitinide, thiazolidinedione, sulfonylurea, dipeptidyl peptidase 4 [DPP4] inhibitors and sodium-glucose co-transporter-2 [SGLT-2] inhibitors), insulin and glucagon-like peptide-1 receptor agonists were documented during the study period according to pharmacological and Anatomical Therapeutic Chemical (ATC) classification.

### Geriatric assessment

Cognitive function using the mini-mental state examination (MMSE) was assessed through the Chinese version of the questionnaire. Patient nutritional status was evaluated by the Mini Nutritional Assessment (MNA) [19]. Trunk balance and core activity were measured by the timed up and go (TUG) test using a 46-cm-height armchair and involved regular footwear, any mobility aids, walking a straight line for 3 m, turning around, walking back to the chair, and sitting down [18, 20, 21]. Mobility and slowness were determined by the 6-meter walking (6 MW) test in which patients were instructed to walk at their self-selected usual pace on a smooth, horizontal walkway [22]. Handgrip strength (HGS) involving the dominant hand was measured and recorded three times, with the maximum value determined by a dynamometer (Smedley’s Dynamometer, TTM, Tokyo, Japan). Rather than using traditional parameters for physical functionality [23, 24], categorized cutoff points were used to define the frailty parameters, including TUG, HGS and the 6-meter walking test (6 MW) [18, 20, 25]. TUG values were separated into tertiles (T1, 0 ~ < 14 s; T2, ≥ 14 ~ < 21 s; T3, ≥ 21 s), while the Chi-square test was used to determine the appropriateness of 21 s. The HGS values were divided into fifth (F1, 0 ~ 15.47 kg; F2, > 15.47 ~ < 20.4 kg; F3, ≥ 20.4 ~ 22.73 kg; F4, > 22.73 ~ 26.9 kg; F5 > 26.9 ~ 48.87 kg in men; F1, 0 ~ < 10.57 kg; F2, ≥ 10.57 ~ < 12.5 kg; F3, ≥ 12.5 ~ 14.83 kg; F4, > 14.83 ~ 17.43 kg; F5, > 17.43 ~ 24.1 kg in women), with abnormal HGS being defined as less than the cut-off points of 20.4 kg for men and 10.57 kg for women.

Abnormal values of 6 MW were separately calculated as quartiles (Q1, 0 ~ 8.95 s; Q2, > 8.95 ~ 12.7 s; Q3, ≥ 12.7 ~ 16.6 s; Q4, > 16.6 ~ 52.0 s in men; Q1, 0 ~ < 8.0 s; Q2, ≥ 8.0 ~ 11.8 s; Q3, > 11.8 ~ 17.51 s; Q4, > 17.51 ~ 50.0 s in women), with cutoff points of > 8.95 s for men and > 17.51 s for women, due to men and women walking at different speeds because of different leg lengths.

### Calculation of frailty

Both the Fried phenotypic model [26] and the Rockwood frailty index [27] are currently used to define frailty according to the Asia-Pacific clinical practice guidelines [23]. Cumulative health deficits have examined the association between frailties, as defined by the Rockwood frailty index [27]. A modified Rockwood frailty index (RFI) defining cross-cutting risk factors was used to measure frailty by utilizing cumulative multi-dimensional health deficits collected in health assessments, including four items of CGA (MNA-SF, TUG, HGS, and 6 MW), 20 chronic diseases except for DM and CKD, and 19 abnormal laboratory data. Categorization of the modified RFI was determined according to established cutoffs in community-dwelling cohorts to match the Fried physical phenotype: non-frail (0–0.1), pre-frail (> 0.1–0.21), and frail (> 0.21) [28]; however, these categories were not good enough to predict the outcome. Therefore, a Rockwood frailty index ≥ 0.313 for outcome prediction was assessed by Area under the Receiver Operating Characteristic (ROC) curve (AUC) under the nonparametric assumption with 68.8% accuracy, 25.4% positive predictive values and 95.0% negative predictive values.

### Study outcome, ascertainment of OAD use, and follow-up

The primary outcome of this study was all-cause mortality. All-cause death was determined based on the Clinical Information Research and Development Center, TCVGH, with the accuracy of death being validated by Taiwan’s National Death Registry, according to either ICD-9 (ICD9 001.x-999.x) or ICD10 (A00.x-Z99.x). The index date was the date of DM and CKD diagnosis. CGA was completed around the time of DM diagnosis. Ascertainment of OAD exposure was defined as the cumulative use of at least one type of medicine for ≥ 90 days before and after the index date; a strategy utilized by other pharmacoepidemiology studies [29]. All participants were followed up until either death or June 19, 2018 to prevent lead-time bias.

### Statistical analyses

Statistical analyses were performed with SPSS for Windows version 22.0 (SPSS Institute Inc., Chicago, USA). For continuous variables in the baseline characteristics, we used the Kolmogorov-Smirnov test to determine the normality of sample distributions. Continuous variables were analyzed by Mann-Whitney *U* tests, generating the median and interquartile range (IQR). Categorical variables, presented as numbers and percentages, were tested by Chi-square or Fisher’s exact tests, followed by Bonferroni post hoc analysis for multiple testing. During the follow-up period, we used Kaplan-Meier analyses to examine cumulative survival, with a comparison made between with and without CKD groups using the log-rank test. Subgroup analyses in the Kaplan-Meier (KM) plots were generated to compare various cumulative survival rates in different subgroups by the log-rank (Mantel-Cox), as well as pairwise comparisons to evaluate the effect of CKD, frailty and different physical function on long-term mortality. The combined assessment of a previously defined high RFI, CKD or not, and high or low functioning status was delineated between KM analyses and Cox proportional hazard models for predicting clinical outcomes in older adults with diabetes. In addition, Cox proportional hazard models were used to evaluate the effects of CKD, frailty, nutrition, TUG, 6 MW and HGS on long-term mortality, independent of the roles exerted by age, gender and the Charlson comorbidity index. P values for nonlinearity were calculated using the null hypothesis test. Statistical significance was set at *P* < 0.05.

## Results

### Baseline characteristics of patients

For the 921 elderly DM patients, the mean (SD) age was 82.0 ± 6.7 years. Compared with the DM patients without CKD (n = 361), the CKD patients (n = 560) were predominantly male and had a high percentage of hypertension, hyperlipidemia, COPD, low serum hemoglobin (HgB) and low eGFR (Table 1). We found a prevalence of 79.3% frailty and 18.4% pre-frailty in all subjects with diabetes (see Additional file 1: Table S1). Individuals with CKD had a significantly longer TUG test of ≥ 21 s (35.2% vs. 24.8%, *P* = 0.035), higher RFI (median [quartiles] = 0.31 [0.25–0.38] vs. 0.24 [0.17–0.29], *P* < 0.001), a higher percentage of RFI ≥ 0.313 (49.6% vs. 18.8%, *P* < 0.001), and a higher prevalence of frailty (87.3% vs. 59.8%, *P* < 0.001), when compared to DM patients without CKD (Table 1, Additional file 2: Table S2).

**Table 1:** Baseline characteristics of older patients with diabetes without and with chronic kidney disease

Characteristics	DM (n = 921)		DM without CKD (n = 361)		DM with CKD (n = 560)		*P* value
Age (years)	83.2	(78.1–86.9)	82.4	(77.3–86.3)	83.4	(78.4–87.0)	0.085
Male	680	(73.8)	252	(69.8)	428	(76.4)	0.031
BMI (kg/m^2^)	23.9	(21.5–26.6)	23.8	(21.5–26.5)	24.0	(21.5–26.8)	0.583
Comorbidities							
Dementia	286	(31.1)	103	(28.5)	183	(32.7)	0.210
Hypertension	843	(91.5)	309	(85.6)	534	(95.4)	< 0.001
Hyperlipidemia	480	(52.1)	159	(44.0)	321	(57.3)	< 0.001
Heart failure	34	(3.7)	12	(3.3)	22	(3.9)	0.767
Cardiovascular disease	278	(30.2)	105	(29.1)	173	(30.9)	0.610
COPD	403	(43.8)	120	(33.2)	283	(50.5)	< 0.001
CCI	1.0	(1.0–2.0)	1.0	(1.0–2.0)	2.0	(1.0–2.0)	0.362
Geriatric assessment							
MNA-SF (0 to 14)	12.0	(9.5–14.0)	12.0	(9.0–14.0)	12.0	(10.0–14.0)	0.929
TUG test ≥ 21 s (n = 430)	135	(31.4)	39	(24.8)	96	(35.2)	0.035
Poor HGS (F < 10.57/M < 20.4 kg) (n = 309)	125	(40.5)	52	(44.1)	73	(38.2)	0.369
Prolonged 6 MW (F > 17.51/M > 8.95 s) (n = 260)	87	(33.5)	39	(35.1)	48	(32.2)	0.718
Rockwood frailty index	0.28	(0.21–0.35)	0.24	(0.17–0.29)	0.31	(0.25–0.38)	< 0.001
Non-frail (0–0.10)	19	(2.1)	16	(4.4)	3	(0.5)	< 0.001
Pre-frail (> 0.10–0.21)	197	(21.4)	129	(35.7)	68	(12.1)	
Frail (> 0.21)	705	(76.6)	216	(59.8)	489	(87.3)	
Rockwood frailty index ≥0.313	346	(37.6)	68	(18.8)	278	(49.6)	< 0.001
Laboratory data							
HgB (g/dL)	12.3	(10.8–13.5)	12.7	(11.1–13.9)	12.0	(10.6–13.3)	< 0.001
Albumin (g/dL)	3.8	(3.4–4.2)	3.8	(3.4–4.3)	3.8	(3.3–4.2)	0.916
Fasting glucose (mg/dL)	128.0	(102.0-177.3)	131.0	(104.0-179.0)	126.0	(101.0-175.0)	0.262
Hba1c (%)	6.5	(5.9–7.5)	6.6	(5.9–7.5)	6.5	(5.9–7.4)	0.878
LDL-C (mg/dL)	94.0	(74.0-117.0)	95.0	(93.8-116.3)	94.0	(75.0-118.0)	0.953
eGFR (ml/min per 1.73 m^2^)							< 0.001
≥ 90	153	(16.6)	107	(29.6)	46	(8.2)	
60 ≤ eGFR < 90	419	(45.5)	254	(70.4)	165	(29.5)	
30 ≤ eGFR < 60	239	(26.0)	0	(0.0)	239	(42.7)	
15 ≤ eGFR < 30	62	(6.7)	0	(0.0)	62	(11.1)	
eGFR < 15	48	(5.2)	0	(0.0)	48	(8.6)	
Proteinuria (mg/g)	0.18	(0.10–0.57)	0.17	(0.09–0.53)	0.21	(0.12–0.80)	0.118
Medications (n = 454)							
Biguanide (n = 454)	226	(49.8)	103	(66.0)	123	(41.3)	< 0.001
SU (n = 454)	206	(45.4)	76	(48.7)	130	(43.6)	0.349
Insulin (n = 454)	256	(56.4)	84	(53.9)	172	(57.7)	0.490
DPP4i (n = 454)	160	(35.2)	51	(32.7)	109	(36.6)	0.472
Meglitinide (n = 454)	55	(12.1)	11	(7.1)	44	(14.8)	0.025
Thiazolidinedione (n = 454)	35	(7.7)	16	(10.3)	19	(6.4)	0.198
Alpha-glucosidase inhibitors (n = 454)	52	(11.5)	11	(7.1)	41	(13.8)	0.048

Continuous data are expressed as median (IQR, interquartile range) and analyzed by the Mann-Whitney *U* test. Categorical data are expressed as number and percentage and analyzed by the Chi-Square test.* BMI* body mass index,* COPD* chronic obstructive pulmonary disease,* CCI* Charlson Comorbidity Index,* MNA-SF* mini-nutritional assessment-short form,* TUG* timed up and go,* HGS* handgrip strength,* 6 MW,* 6-meter walking,* HgB* hemoglobin,* Hba1c* glycated hemoglobin,* LDL-C* Low-density lipoprotein cholesterol,* eGFR* estimated glomerular filtration rate,* SU* sulphonylurea,* DPP4i* dipeptidyl peptidase-4 inhibitors. The presence of cardiovascular diseases, i.e., coronary artery disease, stroke, congestive heart failure, arrhythmia and peripheral arterial disease was determined using patient medical records. eGFR, calculated by using a modified Modification Diet of Renal Disease (MDRD) formula, was utilized to evaluate renal function.

### Comparison between survivors and deceased older patients with diabetes mellitus (DM)

In terms of the clinical characteristics seen in DM survivors and non-survivors, deceased patients with diabetes had a higher percentage of CKD, higher levels of CKD stages 4 and 5, higher COPD rate, a relatively longer TUG, significantly abnormal HGS, and a significantly higher RFI (median [quartiles] = 0.37 [0.31–0.44] vs. 0.27 [0.21–0.33], Table 2, Additional file 3: Table S3). Additionally, deceased patients with diabetes also had lower levels of HgB, serum albumin and eGFR, but higher levels of fasting glucose and proteinuria. Furthermore, deceased patients with diabetes had significantly poor overall data from the geriatric assessment, including MNA-SF, TUG test, poor HGS, prolonged 6 MW and cumulative health deficits for RFI (Table 2, Additional file 3: Table S3).

**Table 2:** Comparison between survivors and deceased older patients with diabetes with and without chronic kidney disease

	Alive (n = 804)		Dead (n = 117)		*P* value
Age (years)	83.0 (77.9–86.8)		84.4 (78.7–87.6)		0.077
Male	593 (73.8)		87 (74.4)		0.979
Chronic kidney disease	470 (58.5)		90 (76.9)		< 0.001
BMI (kg/m^2^)	24.0 (21.6–26.6)		23.6 (21.1–27.0)		0.534
Comorbidities					
Dementia	254 (31.6)		32 (27.4)		0.412
Hypertension	732 (91.0)		111 (94.9)		0.226
Hyperlipidemia	412 (51.2)		68 (58.1)		0.196
Heart failure^f^	28 (3.5)		6 (5.1)		0.426
Cardiovascular disease	241 (30.0)		37 (31.6)		0.799
COPD	339 (42.2)		64 (54.7)		0.014
CCI	1.0 (1.0–2.0)		2.0 (0.0–3.0)		0.649
Geriatric assessment					
MNA-SF (0 to 14)	12.0 (10.0–14.0)		11.0 (8.3–13.0)		0.002
TUG test ≥ 21 s (n = 430)	117 (29.9)		18 (47.4)		0.041
Poor HGS (F < 10.57/M < 20.4 kg) (n = 309)	107 (38.4)		18 (60.0)		0.036
Prolonged 6 MW (F > 17.51/M > 8.95 s) (n = 260)	87 (35.4)		0 (0.0)		0.003
Rockwood frailty index	0.27 (0.21–0.33)		0.37 (0.31–0.44)		< 0.001
Non-frail (0–0.10)	19 (2.4)		0 (0.0)		< 0.001
Pre-frail (> 0.10–0.21)	193 (24.0)		4 (3.4)		
Frail (> 0.21)	592 (73.6)		113 (96.6)		
Rockwood frailty index ≥ 0.313	258 (32.1)		88 (75.2)		< 0.001
CKD stage					< 0.001
Non-CKD	334 (41.5)		27 (23.1)		
Stage 1–2	187 (23.3)		24 (20.5)		
Stage 3	208 (25.9)		31 (26.5)		
Stage 4	49 (6.1)		13 (11.1)		
Stage 5	26 (3.2)		22 (18.8)		
Laboratory data					
HgB (g/dL)	12.4 (11.1–13.6)		11.0 (9.2–12.6)		< 0.001
Albumin (g/dL)	3.9 (3.4–4.2)		3.4 (2.9–3.8)		< 0.001
Fasting glucose (mg/dL)	126.0 (102.0-172.5)		145.0 (105.5–207.0)		0.009
Hba1c (%)	6.5 (5.9–7.5)		6.6 (5.8–7.4)		0.975
eGFR (ml/min per 1.73 m^2^)	63.8 (45.1–82.2)		41.9 (19.5–69.3)		< 0.001
Proteinuria (mg/g)	0.17 (0.09–0.50)		0.36 (0.14–0.89)		0.002
Medications (*n* = 454)					
Biguanide (*n* = 454)	201 (53.0)		25 (33.3)		0.003
SU (*n* = 454)	175 (46.2)		31 (41.3)		0.521
Insulin (*n* = 454)	202 (53.3)		54 (72.0)		0.004
DPP4i (*n* = 454)	137 (36.2)		23 (30.7)		0.438
Meglitinide (*n* = 454)	45 (11.9)		10 (13.3)		0.873
Thiazolidinedione (*n* = 454)	31 (8.2)		4 (5.3)		0.544
Alpha-glucosidase inhibitors (*n* = 454)	45 (11.9)		7 (9.3)		0.665

Continuous data are expressed as median (IQR, interquartile range) and analyzed by the Mann-Whitney *U* test. Categorical data are expressed as number and percentage and analyzed by the Chi-Square test.* BMI* body mass index,* COPD* chronic obstructive pulmonary disease,* CCI* Charlson Comorbidity Index,* MNA-SF* mini-nutritional assessment-short form,* TUG* timed up and go,* HGS* handgrip strength,* 6 MW* 6-meter walking,* CKD* chronic kidney disease,* HgB* hemoglobin,* Hba1c* glycated hemoglobin,* LDL-C* low-density lipoprotein cholesterol,* eGFR* estimated glomerular filtration rate,* SU* sulphonylurea,* DPP4i* dipeptidyl peptidase-4 inhibitors. eGFR, calculated by using modified the Modification Diet of Renal Disease (MDRD) formula, was utilized to evaluate renal function. The presence of cardiovascular diseases, i.e., coronary artery disease, stroke, congestive heart failure, arrhythmia and peripheral arterial disease was determined using patient medical records

### Risk for mortality among DM patients with and without chronic kidney disease (CKD)

During the follow-up period (median [quartiles] = 2.92 [1.06–4.43] years), a univariate Cox regression model showed that CKD (crude hazard ratio [cHR] = 1.92, 95% CI 1.25–2.95), displayed high Charlson Comorbidity Index (CCI) (cHR = 1.17, 95% CI 1.00–1.37), poor nutrition (cHR = 1.18, 95% CI 1.11–1.23), high categorized values of RFI (≥ 0.313) (cHR = 5.88, 95% CI 3.86–8.96), longer TUG (cHR = 2.23, 95% CI 1.18–4.22) and abnormal HGS (cHR = 2.66, 95% CI 1.28–5.53) were all significantly associated with all-cause mortality. After adjusting for the other confounders, CCI remained a significant mortality risk factor (adjusted hazard ratio [aHR] = 4.64, 95% CI 1.09–19.82) (Additional file 4: Table S4).

In those patients without CKD, a high CCI (cHR = 1.44, 95% CI 1.09–1.90), poor nutrition (cHR = 1.16, 95% CI 1.04–1.30), RFI ≥ 0.313 (cHR = 5.28, 95% CI 2.47–11.26) and high fasting glucose (cHR = 1.01, 95% CI 1.003–1.01) were all significantly associated with all-cause mortality (simple model 1, Table 3). However, high serum albumin and eGFR significantly reduced all-cause mortality (simple model, Table 3). After adjustment for confounders, low serum albumin and high fasting glucose remained a significant risk for all-cause mortality in those patients with DM and without CKD (multiple model 1, Table 3).

**Table 3:** Predictors of all-cause mortality in older patients with diabetes with and without chronic kidney disease

	DM without CKD				DM with CKD								
	Simple model 1		Multiple model 1		Simple model 2			Multiple model 2		Multiple model 3		Multiple model 4	
	HR (95% CI)		HR (95% CI)		HR (95% CI)			HR (95% CI)		HR (95% CI)		HR (95% CI)	
Age	1.00 (0.94–1.06)				1.03 (0.99–1.06)								
Male vs. Female	1.05 (0.46–2.39)				0.97 (0.60–1.58)								
CCI	1.44 (1.09–1.90)*		1.28 (0.89–1.83)		1.08 (0.88–1.32)								
Comprehensive geriatric assessment													
MNA-SF	0.86 (0.77–0.96)**		1.03 (0.83–1.27)		0.85 (0.80–0.91)**			1.00 (0.76–1.31)		0.97 (0.88–1.07)		0.94 (0.86–1.04)	
RFI ≥ 0.313	5.28 (2.47–11.26)**		1.90 (0.54–6.64)		5.92 (3.44–10.18)**			4.01 (0.96–16.78)		4.77 (1.98–11.49)*		5.34 (2.23–12.80)**	
TUG test	1.00 (0.93–1.06)				1.01 (0.99–1.03)								
TUG test ≥ 21 s	1.05 (0.21–5.22)				2.50 (1.22–5.13)*			2.98 (0.84–10.51)					
Prolonged 6 MW	0.02 (0.00-53.10)				0.03 (0.00-9.26)								
Poor HGS	2.82 (0.70-11.31)				2.67 (1.12–6.37)*			1.17 (0.31–4.42)					
Laboratory data													
HgB	0.85 (0.71–1.03)				0.68 (0.60–0.76)**					0.76 (0.65–0.89)**		0.75 (0.64–0.87)**	
Albumin	0.23 (0.13–0.43)**		0.19 (0.07–0.53)**		0.40 (0.31–0.52)**					0.67 (0.40–1.10)			
Fasting glucose	1.01 (1.003–1.01)**		1.004 (1.0001-1.01)*		1.00 (1.00–1.00)								
eGFR	0.98 (0.97-1.00)*		1.00 (0.99–1.02)		0.98 (0.97–0.99)**					0.99 (0.98–0.999)*		0.99 (0.98-1.00)	
Proteinuria	0.99 (0.51–1.90)				1.15 (1.04–1.26)**					0.96 (0.84–1.09)		1.00 (0.88–1.13)	

*P < 0.05; **P < 0.01; Multiple model 1 in DM without CKD: The Cox proportional hazard model was used to evaluate the association of all-cause mortality with multivariate analysis among the Charlson Comorbidity Index (CCI), mini-nutritional assessment-short form (MNA-SF), Rockwood frailty index (RFI) ≥ 0.313, serum albumin, glucose, and eGFR. Multiple model 2 in DM with CKD: The Cox proportional hazard model was used to evaluate the association of all-cause mortality with multivariate analysis among MNA-SF, RFI ≥ 0.313, timed up and go (TUG) test ≥ 21 s, and abnormal handgrip strength (HGS). Multiple model 3 in DM with CKD: The Cox proportional hazard model was used to evaluate the association of all-cause mortality with multivariate analysis among MNA-SF, RFI ≥ 0.313, serum HgB, albumin, eGFR, and proteinuria. Multiple model 4 in DM with CKD: The Cox proportional hazard model was used to evaluate the association of all-cause mortality with multivariate analysis among MNA-SF, RFI ≥ 0.313, serum HgB, eGFR, and proteinuria. Prolonged 6 MW (F > 17.51/M > 8.95 s); Poor HGS (F < 10.57/M < 20.4 kg).* 6 MW* 6-meter walking test,* HGS* handgrip strength,* eGFR* estimated glomerular filtration rate,* eGFR* calculated by using modified Modification diet of renal disease (MDRD) formula, was utilized to evaluate renal function.

Among older adults with diabetes and CKD, poor nutrition (cHR = 1.18, 95% CI 1.10–1.25), RFI ≥ 0.313 (cHR = 5.92, 95% CI 3.44–10.18), longer TUG (cHR = 2.50, 95% CI 1.22–5.13), abnormal HGS (cHR = 2.67, 95% CI 1.12–6.37) and proteinuria (cHR = 1.15, 95% CI 1.04–1.26) were all significantly associated with all-cause death (simple model 2, Table 3). However, high HgB, serum albumin and eGFR significantly reduced all-cause mortality (simple model 2, Table 3). After adjustment, high HgB (adjusted HR [aHR] = 0.75, 95% CI 0.64–0.87) and RFI ≥ 0.313 (aHR = 5.34, 95% CI 2.23–12.80) were still significantly associated with all-cause death (multiple model 4, Table 3).

### Survival curves in subgroup analyses of CKD, frailty, and different levels of handgrip strength (HGS) and timed up and go (TUG) in elderly DM patients

**Fig. 2:**
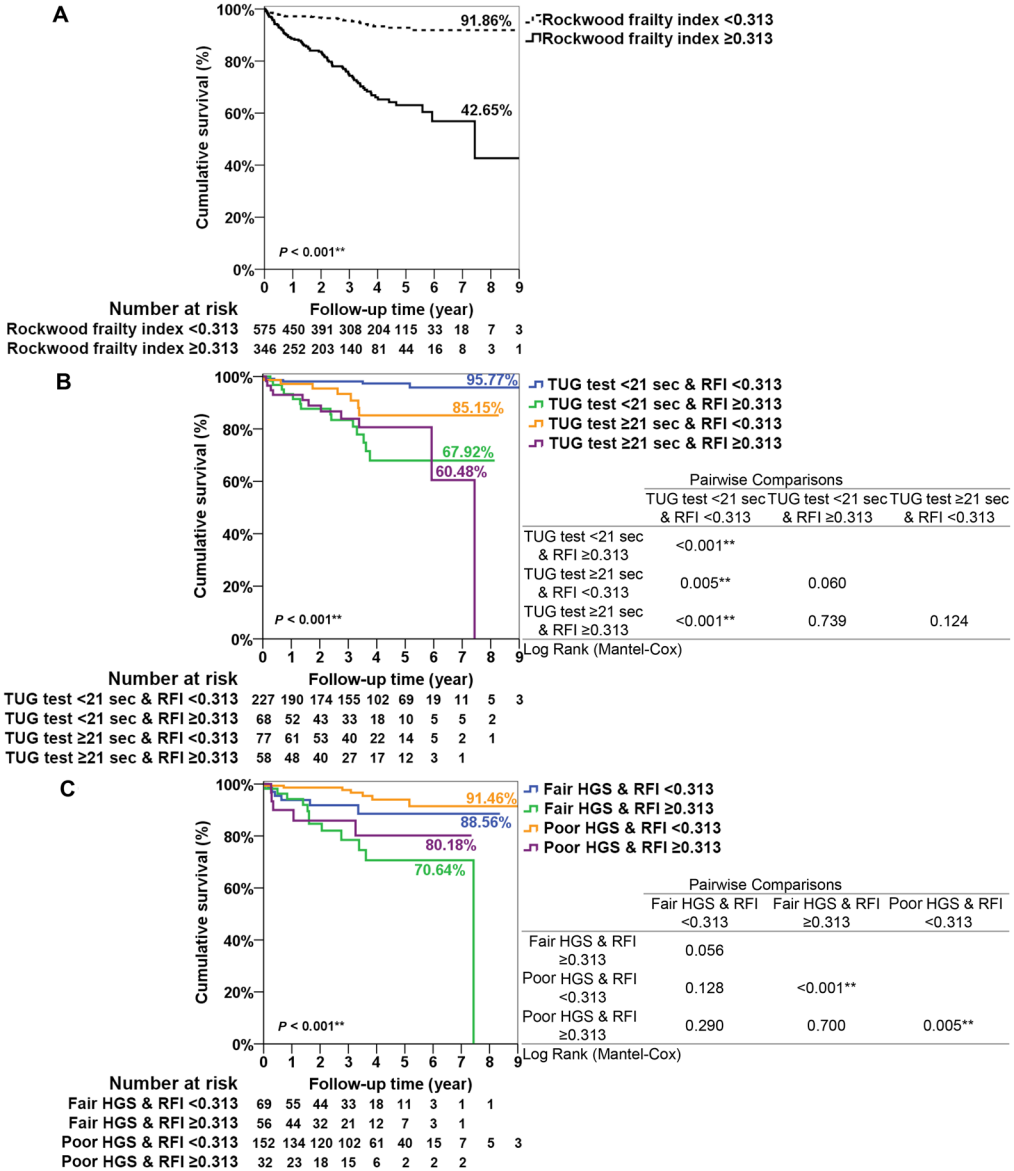
** A** Kaplan-Meier survival curves for mortality stratified by different levels of the Rockwood frailty index. **B** Kaplan-Meier survival curves for mortality stratified by the short or long TUG and different levels of frailty in DM older adults. **C** Kaplan-Meier survival curves for mortality stratified by fair or poor HGS and different levels of frailty in DM older adults. *DM* diabetes mellitus, *CKD* chronic kidney disease, *RFI* Rockwood frailty index, *TUG* timed up and go test, *HGS* handgrip strength. Poor HGS in women < 10.57 kg and men < 20.4 kg; Fair HGS in women ≥ 10.57 kg and men ≥ 20.4 kg. **P < 0.01

In patients with diabetes and an RFI ≥ 0.313, the survival proportion was 42.7%, lower than the 91.9% seen in patients with an RFI < 0.313 (Fig. 2A). Furthermore, the combined impact of an RFI ≥ 0.313 and a prolonged TUG ≥ 21 s resulted in the poorest survival rate (60.5%), followed by TUG < 21 s and RFI ≥ 0.313 (67.9%), TUG ≥ 21 s and RFI < 0.313 (85.2%), and TUG < 21 s and RFI < 0.313 (95.8%) (*P* < 0.001, Fig. 2B). DM older adults with fair HGS but an RFI ≥ 0.313 had the poorest survival rate (70.6%), followed by poor HGS and RFI ≥ 0.313 (80.2%), fair HGS and RFI < 0.313 (88.6%), and poor HGS but RFI < 0.313 (91.5%) (*P* < 0.001, Fig. 2C).

**Fig. 3:**
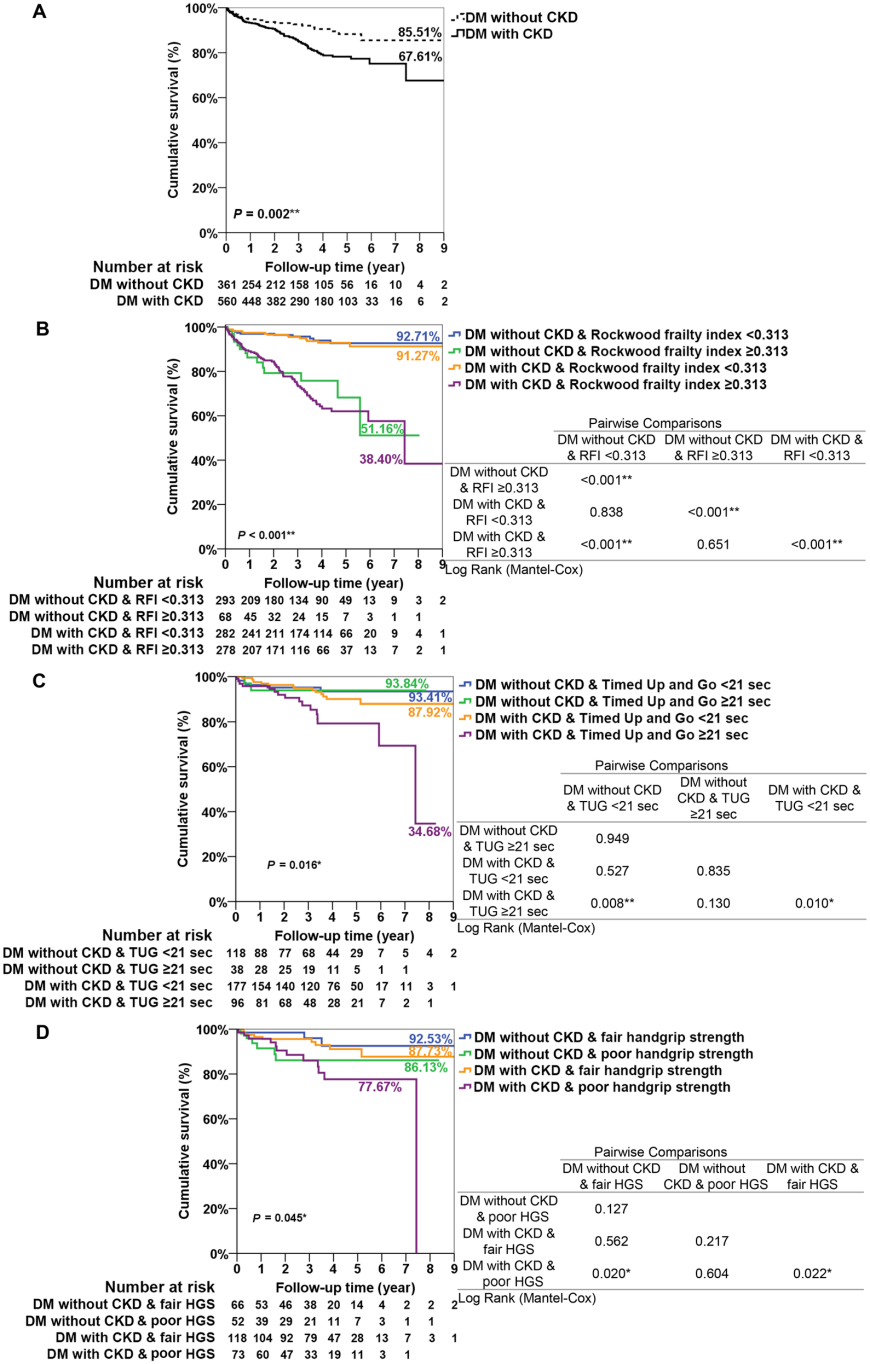
** A** Kaplan-Meier survival curves for mortality stratified by the presence and absence of CKD. **B** Kaplan-Meier survival curves for mortality stratified by the presence or absence of CKD and different levels of frailty in DM older adults. **C** Kaplan-Meier survival curves for mortality stratified by the presence or absence of CKD with a short or long TUG test in DM older adults. **D** Kaplan-Meier survival curves for mortality stratified by the presence or absence of CKD with fair or poor HGS in DM older adults. *DM* diabetes mellitus, *CKD* chronic kidney disease, *RFI* Rockwood frailty index, *TUG* timed up and go test, *HGS* handgrip strength. Poor HGS in women < 10.57 kg and men < 20.4 kg; Fair HGS in women ≥ 10.57 kg and men ≥ 20.4 kg. *P < 0.05, **P < 0.01

We found that elderly DM patients with CKD experienced a lower cumulative survival rate (67.6%) than those without CKD (85.5%) after an approximately 9-year follow-up period (*P* = 0.002, Fig. 3A). In consideration of CKD and RFI, elderly DM patients with CKD and an RFI ≥ 0.313 had the poorest survival rate (38.4%), followed by without CKD and RFI ≥ 0.313 (51.2%), CKD and RFI < 0.313 (91.3%), and without CKD and RFI < 0.313 (92.7%) (*P* < 0.001, Fig. 3B). Regarding CKD and TUG, the older adults with diabetes and CKD and a TUG period ≥ 21 s had the poorest survival rate (34.7%), followed by CKD and TUG < 21 s (87.9%), without CKD and TUG < 21 s (93.4%), and without CKD and TUG ≥ 21 s (93.8%) (*P* = 0.016, Fig. 3C). Finally, elderly DM patients with CKD and a poor handgrip strength (women < 10.57 kg and men < 20.4 kg) experienced the poorest survival rate (77.7%), followed by without CKD and poor HGS (86.1%), CKD and fair HGS (87.7%), and without CKD and fair HGS (92.5%) (*P* = 0.045, Fig. 3D).

## Discussion

The main finding of this study was that older patients with diabetes and CKD experienced a higher prevalence of frailty. In addition, sub-analysis revealed that mortality is significantly associated with CKD, higher Rockwood frailty scores and TUG values, and lower HGS. Furthermore, it was shown that in older patients with diabetes there were additive effects between CKD, frailty, poor HGS and longer TUG concerning survival.

Frailty prevalence is higher in patients with diabetes than it is in nondiabetics [7]. Possible mechanisms for this include neuropathic and inflammatory mechanisms, lower activity of anabolic hormones, as well as resistance to insulin by skeletal muscles, and hyperglycemia, which may accelerate muscle loss and sarcopenia, and in turn, physical function [30, 31]. In addition to micro and macrovascular complications, diabetes may also have an impact on physical, cognitive and functional consequences, thus possibly contributing to the development of frailty [31]. In our study, among the participants with CKD, poor HGS and prolonged TUG values were seen when compared to those without CKD. This finding was in line with several previous studies and showed the association between frailty, physical performance and worsening kidney function [12, 13, 32]. It was proposed that along with a decline in renal function, insulin resistance, chronic inflammation and vascular calcification can all lead to a loss of musculoskeletal mass, and consequently mobility limitation [12, 13, 32].

Traditionally, in patients with diabetes, death is attributed to many classical risk factors, including hypertension, dyslipidemia, smoking and cardiovascular disease in approximately 60% of the patients [1]. On the other hand, a previous study reported that frailty is also a prognostic factor for mortality, albeit independent of diabetes-related complications, and is now considered an important predictor of vital prognosis [9]. In patients with diabetes experiencing frailty, it has been proposed that a combination of factors, such as falls, severe hypoglycemia and higher hospitalization rates, together contribute to the relationship of frailty with mortality. Furthermore, several studies have reported that CKD associated with physical function decline can further worsen mortality in CKD patients [12, 13, 32]. In our study, the HR regarding mortality in those patients with diabetes and a higher Rockwood frailty index was higher. Moreover, in patients with a lower frailty index, even with CKD, their length of survival was not different from that of patients without CKD (Additional file 5: Table S5). It was determined that those with CKD and an increased frailty index experienced the highest mortality rate. This finding was compatible with previous studies which had reported that frailty increases the risk of all-cause mortality in CKD patients [12, 13]. CKD patients, due to their having reduced protein reserves and body energy, experience an increased risk of frailty and a decrease in strength, which in turn can lead to difficulties in their self-care abilities. In addition, a higher frailty index may represent more cumulative deficits in various domains of health (e.g., multimorbidity), which is common in patients with diabetes [33]. Overall, based on many reasons; mortality risk can be amplified by frailty in elderly patients with diabetes, particularly when it is combined with CKD. Further research is still required to better determine the exact mechanisms by which frailty increases mortality risk in DM patients with CKD.

Previous studies have shown that TUG predicts all-cause mortality in older adults [34]. Additionally, it has also been shown in CKD patients that each 1-second increase in TUG is associated with an 8% higher risk for death [35]. A poor TUG performance has been linked to recurrent falls, impaired physical and cognitive function, poor quality of life, dementia and frailty [36]. Additionally, CKD patients experienced a higher prevalence of clinical and subclinical multisystem comorbidities and vascular dysfunction. Thus, a slow walking speed may reflect the cumulative multisystem comorbid burden associated with CKD and mortality risk. In our study, abnormal TUG results were associated with higher all-cause mortality in older patients with diabetes and CKD than those without CKD. Those with CKD and slower TUG results experienced the highest mortality rate. These findings suggest that CKD and physical performance in older adults with diabetes may share common risk factors and disease mechanisms. This present analysis also showed that greater handgrip strength (HGS) is associated with a significantly lower incidence of death; results that were compatible with a previous study of patients with diabetes [37–39]. Furthermore, the association between HGS and mortality persisted after an adjustment for confounders, including kidney function and other diabetic complications. Those patients with CKD and lower HGS experienced the highest mortality rate, and there are a variety of possible explanations which may account for the relationship between HGS and outcomes. Most obviously, individuals with higher HGS may be healthier overall than those participants with lower HGS, possibly due to regularly scheduled small resistance training and leisure time physical activity [39].

Based on our findings, early frailty screening, in addition to the evaluation of diabetic vascular complications, is considered an important aspect of the comprehensive care of patients with diabetes [40]. Exercise training, as well as adequate protein and calorie intake, are all necessary for both maintaining and increasing muscle mass [41]. However, there were limitations in our study that warrant consideration. First, the association between kidney function and prevalent frailty was cross-sectional, and causality could not be established as hypertension and heart failure may be mediators. Second, our study focused on older adults in hospitals, whereas previously mentioned studies were on community-dwelling older individuals. Generalization with regards to the other groups is hence uncertain. Additionally, we examined the incidence of all-cause mortality, and detailed analysis surrounding the cause of death was not analyzed. Third, once measured eGFR may not exactly account for some of the observed associations between renal function and frailty. Finally, several factors relevant to frailty, including serum vitamin D levels and vitamin D intake, body composition, sarcopenia, falls and fractures, as well as socioeconomic status, were not assessed in this study. Further prospective studies remain necessary to better elucidate the exact longitudinal relationship between renal function, physical performance and risk of mortality in elderly patients with diabetes.

## Conclusion

In summary, we demonstrated that in hospital-based older adults with diabetes, a diminished eGFR was associated with a higher frailty index, coinciding with diminished walking speed and handgrip strength. Furthermore, we determined that renal function and physical performance were associated with incidents of all-cause mortality, and the combined effects of these factors can be seen in patient outcomes. Our findings highlight the importance of both considering and assessing the possibility of frailty in older adults with diabetes and CKD. Diabetes-related mortality and incidents of disability may be reduced through early intervention involving frailty diagnosis, and subsequently working in conjunction with CKD prevention.

## Supplementary Information

**Additional file 1: Table S1.** Prevalence of frailty and pre-frailty each year in older adults with diabetes.

**Additional file 2: Table S2.** Baseline characteristics of older patients with diabetes with and without chronic kidney disease.

**Additional file 3: Table S3.** Comparison between survivors and deceased older patients with diabetes with and without chronic kidney disease.

**Additional file 4: Table S4.** Predictors of all-cause mortality in older patients with diabetes.

**Additional file 5: Table S5.** Comparison between survivors and deceased older patients with diabetes without and with chronic kidney disease.

## Data Availability

The full data used to support the findings of this study are available from the corresponding author upon request.

## Abbreviations

CGA: Comprehensive geriatric assessment
CKD: Chronic kidney disease
COPD: Chronic obstructive pulmonary disease
ESRD: End-stage renal disease
eGFR: Estimated glomerular filtration rate
DM: Diabetes mellitus
ICD-9-CM: International Classification of Disease 9th version, Clinical Modification
ACR: Albumin/creatinine ratio
PC: Protein/creatinine
CHF: Chronic heart failure
HF: Heart failure
NT-proBNP: N-terminal pro-B-type natriuretic peptide
ATC: Anatomical Therapeutic Chemical
OADs: Oral antidiabetic agents
DPP4: Dipeptidyl peptidase 4
SGLT-2: Sodium-glucose co-transporter-2
MMSE: Mini-mental state examination
MNA: Mini Nutritional Assessment
TUG: Timed up and go
6 MW: 6-meter walking
HGS: Handgrip strength
RFI: Rockwood frailty index
ROC: Receiver Operating Characteristic
AUC: Area under the Receiver Operating Characteristic (ROC) curve
KM: Kaplan-Meier
