# Supplementary material for: Effect of frailty, physical performance, and chronic kidney disease on mortality in older patients with diabetes : a retrospective longitudinal cohort study
Source: Diabetol Metab Syndr. 2023 Jan 17;15:7. doi: 10.1186/s13098-022-00972-0 (PMC9843852; doi:10.1186/s13098-022-00972-0)
Supplement: Supplementary file 1 — Additional file 1: Table S1. Prevalence of frailty and pre-frailty each year in older adults with diabetes. [file 13098_2022_972_MOESM1_ESM.docx]

**Effect of frailty, physical performance, and chronic kidney disease on mortality in older patients with diabetes : a retrospective** **longitudinal** **cohort study**

**Shuo-Chun Weng^1,2,3,4^, Cheng-Fu Lin^2,3,4,5^, Chiann-Yi Hsu^6^ and Shih-Yi Lin^2,4,7*^**

**Additional** **file 1:** **Table S1.** Prevalence of frailty and pre-frailty each year in older adults with diabetes.

| Year | | Number | Percentage |
| --- | --- | --- | --- |
| 2009 | 0-0.1 non-frail | 0 | 0.0% |
|  | >0.1-0.21 pre-frail | 3 | 17.6% |
|  | >0.21 frail | 14 | 82.4% |
|  | Sum | 17 | 100.0% |
| 2010 | 0-0.1 non-frail | 0 | 0.0% |
|  | >0.1-0.21 pre-frail | 5 | 10.2% |
|  | >0.21 frail | 44 | 89.8% |
|  | Sum | 49 | 100.0% |
| 2011 | 0-0.1 non-frail | 0 | 0.0% |
|  | >0.1-0.21 pre-frail | 7 | 15.6% |
|  | >0.21 frail | 38 | 84.4% |
|  | Sum | 45 | 100.0% |
| 2012 | 0-0.1 non-frail | 3 | 1.9% |
|  | >0.1-0.21 pre-frail | 40 | 25.2% |
|  | >0.21 frail | 116 | 73.0% |
|  | Sum | 159 | 100.0% |
| 2013 | 0-0.1 non-frail | 6 | 2.2% |
|  | >0.1-0.21 pre-frail | 67 | 24.7% |
|  | >0.21 frail | 198 | 73.1% |
|  | Sum | 271 | 100.0% |
| 2014 | 0-0.1 non-frail | 5 | 2.5% |
|  | >0.1-0.21 pre-frail | 46 | 23.2% |
|  | >0.21 frail | 147 | 74.2% |
|  | Sum | 198 | 100.0% |
| 2015 | 0-0.1 non-frail | 2 | 1.5% |
|  | >0.1-0.21 pre-frail | 21 | 15.9% |
|  | >0.21 frail | 109 | 82.6% |
|  | Sum | 132 | 100.0% |
| 2016 | 0-0.1 non-frail | 1 | 4.8% |
|  | >0.1-0.21 pre-frail | 2 | 9.5% |
|  | >0.21 frail | 18 | 85.7% |
|  | Sum | 21 | 100.0% |
| 2017 | 0-0.1 non-frail | 2 | 10.0% |
|  | >0.1-0.21 pre-frail | 4 | 20.0% |
|  | >0.21 frail | 14 | 70.0% |
|  | Sum | 20 | 100.0% |
| 2018 | 0-0.1 non-frail | 0 | 0.0% |
|  | >0.1-0.21 pre-frail | 2 | 22.2% |
|  | >0.21 frail | 7 | 77.8% |
|  | Sum | 9 | 100.0% |
