# Supplementary material for: Effect of frailty, physical performance, and chronic kidney disease on mortality in older patients with diabetes : a retrospective longitudinal cohort study
Source: Diabetol Metab Syndr. 2023 Jan 17;15:7. doi: 10.1186/s13098-022-00972-0 (PMC9843852; doi:10.1186/s13098-022-00972-0)
Supplement: Supplementary file 2 — Additional file 2: Table S2. Baseline characteristics of older patients with diabetes with and without chronic kidney disease. [file 13098_2022_972_MOESM2_ESM.docx]

| **Additional file 2: Table S2.**  Baseline characteristics of older patients with diabetes with and without chronic kidney disease. | | | | | | | |
| --- | --- | --- | --- | --- | --- | --- | --- |
| **Characteristics** | **DM**  **(n = 921)** | | **DM without CKD**  **(n = 361)** | | **DM with CKD**  **(n = 560)** | | ***P*-value** |
| **Geriatric assessment** |  |  |  |  |  |  |  |
| Timed up and go test (sec) (n = 430) | 17.0 | (13.0-23.3) | 16.0 | (12.0-20.5) | 18.0 | (14.0-24.0) | 0.082 |
| Handgrip strength (kg) – female (n = 62) | 14.0 | (11.5-16.6) | 14.3 | (11.2-16.4) | 13.5 | (11.5-17.5) | 0.910 |
| Handgrip strength (kg) – male (n = 247) | 21.3 | (16.3-25.4) | 20.0 | (15.4-25.4) | 21.8 | (17.8-25.7) | 0.148 |
| 6-meter walking test (sec) (n = 260) | 13.0 | (9.0-17.1) | 12.7 | (8.5-16.0) | 13.0 | (9.7-18.0) | 0.517 |
| Categorical data are expressed as number and percentage and analyzed by the Chi-square test. | | | | | | | |
