# Supplementary material for: Effect of frailty, physical performance, and chronic kidney disease on mortality in older patients with diabetes : a retrospective longitudinal cohort study
Source: Diabetol Metab Syndr. 2023 Jan 17;15:7. doi: 10.1186/s13098-022-00972-0 (PMC9843852; doi:10.1186/s13098-022-00972-0)
Supplement: Supplementary file 3 — Additional file 3: Table S3. Comparison between survivors and deceased older patients with diabetes with and without chronic kidney disease. [file 13098_2022_972_MOESM3_ESM.docx]

| **Additional file 3: Table S3.** Comparison between survivors and deceased older patients with diabetes with and without chronic kidney disease. | | | | | |
| --- | --- | --- | --- | --- | --- |
|  | **Alive (n = 804)** | | **Dead (n = 117)** | | ***P-*value** |
| **Geriatric assessment** |  |  |  |  |  |
| Timed up and go test (sec) (n = 430) | 17.0 | (13.0-23.0) | 20.0 | (15.8-26.3) | 0.036 |
| Handgrip strength (kg) – female (n = 62) | 14.1 | (11.6-16.9) | 7.8 | (2.9-15.2) | 0.069 |
| Handgrip strength (kg) – male (n = 247) | 21.5 | (16.4-25.6) | 19.6 | (15.2-24.5) | 0.257 |
| 6-meter walking test (sec) (n = 260) | 12.9 | (8.6-17.0) | 13.5 | (11.4-18.8) | 0.269 |

Categorical data are expressed as number and percentage and analyzed by the Chi-square test.
