# Supplementary material for: Effect of frailty, physical performance, and chronic kidney disease on mortality in older patients with diabetes : a retrospective longitudinal cohort study
Source: Diabetol Metab Syndr. 2023 Jan 17;15:7. doi: 10.1186/s13098-022-00972-0 (PMC9843852; doi:10.1186/s13098-022-00972-0)
Supplement: Supplementary file 4 — Additional file 4: Table S4. Predictors of all-cause mortality in older patients with diabetes. [file 13098_2022_972_MOESM4_ESM.docx]

| **Additional file 4: Table S4.** Predictors of all-cause mortality in older patients with diabetes. | | | | | | | |
| --- | --- | --- | --- | --- | --- | --- | --- |
|  | **DM** | | | | | | |
|  | **Simple model 1** | | **Multiple model 1** | | **Multiple model 2** | | |
|  | HR | (95% CI) | HR | (95% CI) | HR | (95% CI) | |
| Age (years) | 1.02 | (0.99-1.05) |  |  |  |  | |
| Male vs. Female | 1.04 | (0.69-1.57) |  |  |  |  | |
| Chronic kidney disease | 1.92 | (1.25-2.95)** | 0.63 | (0.06-6.22) |  |  | |
| Charlson Comorbidity Index | 1.17 | (1.00-1.37)* | 4.64 | (1.09-19.82)* | 0.92 | (0.72-1.18) | |
| **Comprehensive geriatric assessment** | | |  |  |  |  | |
| MNA-SF | 0.85 | (0.81-0.90)** | 3.10 | (0.91-10.57) |  |  | |
| Rockwood frailty index ≥0.313 | 5.88 | (3.86-8.96)** | 5.04 | (0.19-132.47) |  |  | |
| Timed up and go test | 1.01 | (0.99-1.03) |  |  |  |  | |
| TUG test ≥21 sec | 2.23 | (1.18-4.22)* | 6.44 | (0.38-110.52) |  |  | |
| Prolonged 6MW | 0.02 | (0.00-2.37) |  |  |  |  | |
| Poor HGS | 2.66 | (1.28-5.53)** | 4.50 | (0.23-88.14) |  |  | |
| **Laboratory data** |  |  |  |  |  | |  |
| HgB (g/dL) | 0.72 | (0.66-0.80)** |  |  | 0.69 | | (0.56-0.85)** |
| Albumin (g/dL) | 0.36 | (0.28-0.45)** |  |  | 0.50 | | (0.23-1.10) |
| Fasting glucose (mg/dL) | 1.00 | (1.001-1.00)** |  |  | 1.00 | | (1.001-1.01)* |
| eGFR (ml/min per 1.73m^2^) | 0.98 | (0.97-0.99)** |  |  | 0.97 | | (0.96-0.99)** |
| Proteinuria (mg/g) | 1.15 | (1.04-1.26)** |  |  | 0.85 | | (0.65-1.11) |
| **P* < 0.05; ***P* < 0.01; Multiple model 1: The Cox proportional hazard model was used to evaluate the association of all-cause mortality with multivariate analysis among chronic kidney disease, Charlson Comorbidity Index (CCI), mini-nutritional assessment-short form (MNA-SF), Rockwood frailty index ≥0.313, categorized timed up and go test, and abnormal handgrip strength. Multiple model 2: The Cox proportional hazard model was used to evaluate the association of all-cause mortality with multivariate analysis among CCI, serum HgB, albumin, fasting glucose, eGFR, and proteinuria. TUG, timed up and go test; 6MW, 6-meter walking test; HGS, handgrip strength; HgB, hemoglobin; eGFR, estimated glomerular filtration rate. eGFR, calculated by using modified the Modification Diet of Renal Disease (MDRD) formula, was utilized to evaluate renal function. Prolonged 6MW (F >17.51/M >8.95 sec); Poor HGS (F <10.57/M <20.4 kg). | | | | | | | |
