# Supplementary material for: Effect of frailty, physical performance, and chronic kidney disease on mortality in older patients with diabetes : a retrospective longitudinal cohort study
Source: Diabetol Metab Syndr. 2023 Jan 17;15:7. doi: 10.1186/s13098-022-00972-0 (PMC9843852; doi:10.1186/s13098-022-00972-0)
Supplement: Supplementary file 5 — Additional file 5: Table S5. Comparison between survivors and deceased older patients with diabetes without and with chronic kidney disease. [file 13098_2022_972_MOESM5_ESM.docx]

**Additional file 5: Table S5.** Comparison between survivors and deceased older patients with diabetes without and with chronic kidney disease.

|  | | Alive (n = 804) | | Dead (n = 117) | | *P-*value |
| --- | --- | --- | --- | --- | --- | --- |
|  |  |  |  |  |  |  |
| Group 1^†^ |  |  |  |  |  | < 0.001** |
| Without CKD & non-frail [1] |  | 16 | (1.99%) | 0 | (0.0%) | [1] vs. [2] |
| CKD & non-frail [2] |  | 3 | (0.37%) | 0 | (0.0%) | *P* = 1.000 |
| Without CKD & pre-frail [3] |  | 126 | (15.67%) | 3 | (2.56%) | [3] vs. [4] |
| CKD & pre-frail [4] |  | 67 | (8.33%) | 1 | (0.85%) | *P* = 1.000 |
| Without CKD & frail [5] |  | 192 | (23.88%) | 24 | (20.51%) | [5] vs. [6] |
| CKD & frail [6] |  | 400 | (49.75%) | 89 | (76.07%) | *P* = 0.058 |
| Group 2 (n = 430) |  |  |  |  |  | < 0.001** |
| TUG test <21 sec & RFI <0.313 [1] |  | 221 | (56.38%) | 6 | (15.79%) | [1] vs. [2] |
| TUG test <21 sec & RFI ≥0.313 [2] |  | 54 | (13.78%) | 14 | (36.84%) | *P* < 0.001** |
| TUG test ≥21 sec & RFI <0.313 [3] |  | 70 | (17.86%) | 7 | (18.42%) | [3] vs. [4] |
| TUG test ≥21 sec & RFI ≥0.313 [4] |  | 47 | (11.99%) | 11 | (28.95%) | *P* = 0.151 |
| Group 3 (n = 309) |  |  |  |  |  | 0.002** |
| Fair HGS & RFI <0.313 [1] |  | 63 | (22.58%) | 6 | (20.00%) | [1] vs. [2] |
| Fair HGS & RFI ≥0.313 [2] |  | 44 | (15.77%) | 12 | (40.00%) | *P* = 0.142 |
| Poor HGS & RFI <0.313 [3] |  | 145 | (51.97%) | 7 | (23.33%) | [3] vs. [4] |
| Poor HGS & RFI ≥0.313 [4] |  | 27 | (9.68%) | 5 | (16.67%) | *P* = 0.112 |
| Group 4 (n = 260) |  |  |  |  |  | 0.009** |
| Fair 6MW & RFI <0.313 [1] |  | 104 | (42.28%) | 6 | (42.86%) | [1] vs. [2] |
| Fair 6MW & RFI ≥0.313 [2] |  | 55 | (22.36%) | 8 | (57.14%) | *P* = 0.289 |
| Prolonged 6MW & RFI <0.313 [3] |  | 74 | (30.08%) | 0 | (0.0%) | [3] vs. [4] |
| Prolonged 6MW & RFI ≥0.313 [4] |  | 13 | (5.28%) | 0 | (0.0%) | *P* = 1.000 |

^†^Frailty and non-frail older patients were classified according to the Rockwood frailty index. We used the Chi-Square test followed by Bonferroni post hoc analysis for multiple testing. **P* < 0.05, ***P* < 0.01. CKD, chronic kidney disease; TUG, timed up and go test; RFI, Rockwood frailty index; 6MW, 6-meter walking test; HGS, handgrip strength.
